# Effectiveness of Holistic Interventions for People with Severe Chronic Obstructive Pulmonary Disease: Systematic Review of Controlled Clinical Trials

**DOI:** 10.1371/journal.pone.0046433

**Published:** 2012-10-23

**Authors:** Ulugbek Nurmatov, Susan Buckingham, Marilyn Kendall, Scott A. Murray, Patrick White, Aziz Sheikh, Hilary Pinnock

**Affiliations:** 1 Allergy and Respiratory Research Group, Centre for Population Health Sciences, The University of Edinburgh, Edinburgh, Scotland, United Kingdom; 2 Primary Palliative Care Research Group, Centre for Population Health Sciences, The University of Edinburgh, Edinburgh, Scotland, United Kingdom; 3 Department of Primary Care and Public Health Sciences, King's College London, London, England, United Kingdom; University of Ottawa, Canada

## Abstract

**Background:**

Despite a well-recognised burden of disabling physical symptoms compounded by co-morbidities, psychological distress and social isolation, the needs of people with severe chronic obstructive pulmonary disease (COPD) are typically poorly addressed.

**Aim:**

To assess the effectiveness of interventions designed to deliver holistic care for people with severe COPD.

**Methods:**

We searched 11 biomedical databases, three trial repositories (January 1990-March 2012; no language restrictions) and contacted international experts to locate published, unpublished and in-progress randomised controlled trials (RCTs), quasi-RCTs and controlled clinical trials (CCTs) that investigated holistic interventions to support patients with severe COPD in any healthcare context. The primary outcome was health-related quality of life (HRQoL). Quality assessment and data extraction followed Cochrane Collaboration methodology. We used a piloted data extraction sheet and undertook narrative synthesis.

**Results:**

From 2,866 potentially relevant papers, we identified three trials: two RCTs (from United States and Australia), and one CCT (from Thailand): total 216 patients. Risk of bias was assessed as moderate in two studies and high in the third. All the interventions were led by nurses acting in a co-ordinating role (e.g. facilitating community support in Thailand, providing case-management in the USA, or co-ordinating inpatient care in Australia). HRQoL improved significantly in the Thai CCT compared to the (very limited) usual care (p<0.001), in two sub-domains in the American trial, but showed no significant changes in the Australian trial. Exercise tolerance, dyspnoea, and satisfaction with care also improved in the Thai trial.

**Conclusions:**

Some 15 years after reports first highlighted the unmet needs of people with severe COPD, we have been unable to find robust trial evidence about interventions that can address those needs. There is an urgent need to develop and evaluate holistic care interventions designed improve HRQoL for people with severe COPD.

**Systematic Review Registration:**

PROSPERO (CRD42012002430).

## Introduction

Globally, long-term conditions such as chronic obstructive pulmonary disease (COPD) are responsible for increasing morbidity and an increasing proportion of deaths. [1] People with severe COPD have a well-recognised burden of disabling physical symptoms (especially breathlessness), psychological distress and social isolation, [2], [3], [4], [5], [6] often more severe than people with lung cancer. [2] Despite this the clinical and social care needs of these patients, usually compounded by co-morbidities, are typically poorly addressed; [2], [3] leading to calls for equity of access to supportive care services. [7], [8]

The current approach is to build on existing cancer-based palliative care services, [7], [9] which are predicated on an ability to recognise a terminal phase, [10], [11] when physical, psychological, social and spiritual needs can be assessed and holistic care planned. [11], [12] Despite defined prognostic indicators, [13], [14] accurate prognostication for individuals with COPD remains extremely difficult, [12], [15] raising concerns that inability to identify people at risk of dying may act as a barrier to provision of care. [16] A further challenge is the recognised tendency for people with COPD to remain ‘silent’ about their (often very considerable) physical and social disabilities. [17] People with end-stage COPD tend to ‘normalise’ their limitations as the result of ‘old age’, [18] about which ‘nothing can be done’[17] and may be slow to acknowledge end of life concerns [19]. ‘Weary resignation’ after years of futile attempts to improve their circumstances, [20] and/or a ‘recalibration’ of expectations [21] as an adaptive coping strategy may contribute to an undemanding acceptance of their circumstances by patients and their family carers.

Our recent multi-perspective longitudinal qualitative study described the lifelong story of COPD. [22] Significantly for the development of models of care towards the end of life, our data suggested that a ‘clear point of transition’ to palliative care was meaningless in a condition with no clear beginning and an unpredictable, unanticipated end. Instead, we concluded that physical, psychological, social and spiritual needs should be sought proactively, and palliation of symptoms and holistic supportive care intensified according to need, without any formal requirement to identify end-stage disease.

We sought systematically to identify and critically appraise published and unpublished clinical trials that assessed the effectiveness of interventions designed to deliver or enhance holistic care (i.e. addressing physical, psychological, social and spiritual needs) compared to usual care in any healthcare system for people with severe COPD.

## Methods

Our systematic review is registered with PROSPERO (CRD42012002430). We made no substantial amendments to the online protocol. [23] We followed the procedures described in the Cochrane Handbook for Systematic Reviews of Interventions [24] and the PRISMA statement for reporting systematic reviews. [25]

### Inclusion criteria

We were interested in randomised controlled trials (RCTs), which investigated interventions designed to deliver or enhance holistic care (defined as addressing needs within at least three of physical, psychological, social or spiritual domains [7]) for patients with severe COPD in any healthcare context. The intervention could be part of a wider intervention (e.g. pulmonary rehabilitation or integrated care) if it satisfied the definition of a holistic intervention and measured an outcome of interest. Definitions of severe COPD were either a forced expiratory volume in one second (FEV_1_) <50%, or significant breathlessness (e.g. an MRC Dyspnoea score of 4 or 5), or an admission with an exacerbation of COPD, or identified by a clinician as being ‘at risk of dying’ from COPD. We also included quasi-RCTs and controlled clinical trials (CCTs) as preliminary searches suggested that there would be very few RCTs. Although our primary outcome of interest was an improvement in (disease-specific or generic) health-related quality of life (HRQoL) we were also interested in other measures of physical, psychological, spiritual and social well-being, health and/or social service resource use.

### Search strategy

We searched 11 international databases for published material: AMED; British Nursing Index; CINAHL; Cochrane Library; DARE; EMBASE; ISI Web of Science; LILACS; MEDLINE; PsycINFO; ZETOC. (The search terms are detailed in Table S1). In addition, we searched Internet-based international trial repositories www.clinicaltrials.gov; www.controlled-trials.com and the UK Clinical Research Network Study Portfolio; and contacted international experts in order to locate unpublished and on-going work (see Table S2). Our searches covered a 22-year period from January 1990 to March 2012. Early work identifying the lack of supportive care services for people with very severe COPD, [6] and the wider agenda of extending palliative care to people with non-malignant disease, [2], [6], [13] date from the late 1990s so we judged that intervention studies would be unlikely before 1990; our preliminary scoping of the literature confirmed this (details available from corresponding author). The bibliographies of all eligible studies were scrutinised to identify possible additional studies. No language restrictions were employed.

### Selection of studies

After initial screening for obviously ineligible papers (e.g letters, abstracts, editorials, pharmaceutical trials, basic science, surveys, observational studies, reviews, discussion pieces etc) a sub-group of abstracts of potentially eligible studies were scrutinised in detail and independently reviewed by two researchers (UN, and SB or MK). Our eligibility criteria defined holistic care as comprising at least three of the four components (physical, psychological, social and spiritual), but in order to ensure that we did not overlook potentially eligible trials because of limited reporting in the abstract, we only required abstracts to mention two of the components. The full texts of all potentially eligible studies were assessed for eligibility against the inclusion criteria by two reviewers (UN, and SB or MK). Any disagreements were resolved through discussion, with HP arbitrating if agreement could not be reached.

### Quality assessment

Methodological quality of included studies was independently assessed (by UN and SB) using the Cochrane Risk of Bias tool (for RCTs and quasi-RCTs) [24] and the Effectiveness and Practice Organisation of Care criteria (for CCTs). [26] Bias was assessed in the domains of: adequate sequence generation; allocation concealment; blinding of participants and personnel; blinding of outcomes; addressing of incomplete data; absence of selective reporting; absence of other sources of bias. Each parameter and the overall study was graded: low, moderate or high risk of bias. Disagreements between the reviewers were resolved by discussion, with arbitration by HP if needed.

### Data extraction

Data were independently abstracted by two reviewers (UN and SB) onto a customised data extraction sheet. We wrote to authors of all the papers to clarify any details relating to the intervention or data which were unclear from the published report. We treated a study with multiple reports as a single study, but drew on all relevant publications.

### Data synthesis

Based on our preliminary scoping work, we anticipated that we would identify a limited number of eligible trials with substantial heterogeneity so that meta-analysis would not be appropriate. We therefore planned to undertake a narrative synthesis by extracting data about the elements of the interventions under the headings of setting, mode of delivery, aspects of holistic care addressed, duration and intensity of components and the effectiveness (or not) of the intervention. Interpretation was facilitated by discussion amongst the multidisciplinary study team.

## Results

**Figure 1:**
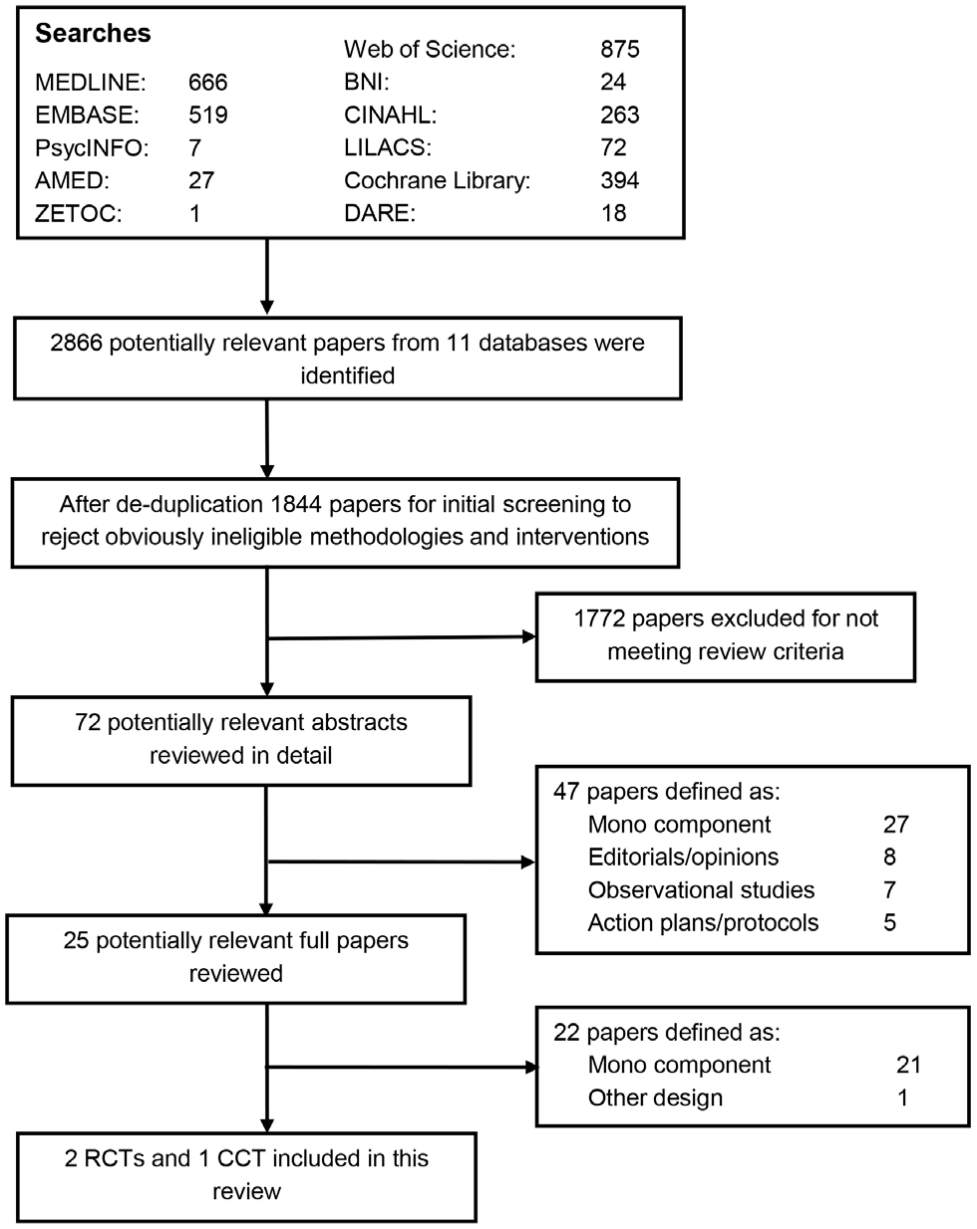
PRISMA flow diagram.

We identified 2,866 potentially relevant publications, from which we identified three papers that satisfied our inclusion criteria (see Figure 1 for the PRISMA flow diagram). We did not identify any unpublished or studies in progress. The responses from the experts whom we contacted did not identify any further studies.

The three studies included a total of 216 people with COPD (see Table 1). Two studies were RCTs [27], [28] (one from USA [27] and one from Australia [28]). One of the RCTs [27] included patients with COPD or chronic heart failure (CHF) and reported sub-group analysis for some of the outcomes: the author responded to our request for further information, but was unable to provide results for any additional outcomes in the COPD group. The third study was a CCT, carried out in Thailand. [29] The authors responded to our request for additional details of the methods.

**Table 1:** Characteristics of included studies.

Study, year	Country, setting	Patient demographics	Description of the delivery of intervention	Aspects of holistic care addressed	Duration and intensity of components	Control
Aiken *et al.*, 2006 [27]	Arizona, USA. Hospice-based community nurse led case management in addition to the patients' usual Managed care Organisation (MCO) services	Hypoxic (oxygen saturation <88% on air) sub-group of COPD patients; with an estimated 2 year life expectancy. Demographics not reported for the COPD sub-group. Study included a total of 192 patients with CHF and COPD: 34 COPD patients received the intervention, 28 controls.	‘PhoenixCare’: Intensive home-based care provided by nurse case managers. Team members included medical director, social worker and pastoral counsellor in association with primary care physician, health plan case manager (if available), patient/family and community agencies.	All four components well addressed. Physical: Medical management, emergency response plan, disease and health education. Psychological and emotional support and counselling. Social Disease and health promotion addressing patient and family understanding of the disease, Community resource referrals. Spiritual Assessment and review of spiritual concerns. Advance Care Planning discussions	Intensive programme of home visits/calls, with an average of 44 contacts ‘over course of the intervention’. Duration of the intervention unclear with most outcomes reported at 3 and 6 months, but some at 9 months. Contacts increased in the event of an exacerbation.	Usual MCO management (which could include ‘usual’ case –management)
Egan *et al.*, 2002 [28]	Brisbane, Australia. Hospital-based, nurse-led case management in a large private hospital	Patients with COPD and/or chronic asthma during an admission to hospital. 48% male, Mean age 67 years. 33 patients received the intervention, 33 controls.	Respiratory nurse-led case management during admission with an exacerbation including assessment on admission, case-conference before discharge, and telephone follow-up at 1 and 6 weeks post-discharge.	All four components well addressed. “A comprehensive nursing assessment identified physical, psychological, social, spiritual and resource needs”	Intense programme of care during admission; with contacts at 1 and 6 weeks after discharge.	Usual inpatient care
Noonill *et al.*, 2007 [29]	Thasala, Thailand.Community-based, nurse led intervention within tambons (administrative sub-districts)	Patients with COPD with no significant co-morbidities and who had a support person willing to participate in the study living nearby. 83% male aged 70 years (SD 6); 44 patients received the intervention, 43 controls	‘Community Care for COPD’. Community nurse-led co-ordination of care focussing on mobilisation of community resources, systematic education, integrating positive health lifestyle changes.	Three components well addressed. Physical: intervention built around concept of pulmonary rehabilitation Psychological: ‘enhanced psychosocial support from the community nurse Social: key focus on mobilising community support (visits from health volunteers and family supervision)	Programme implemented over 12 weeks. Monthly visits by community nurse supplemented by community support including twice-monthly visits from lay community health volunteers as well as family and community supervision	Usual hospital based acute care with ‘limited’ chronic care. ‘Largely inaccessible’ respiratory clinics.

### Description of the interventions

All the interventions were led by nurses acting in a co-ordinating or case-management role, though context and tasks varied. (See Table 1 for details)

- Aiken *et al.*, [27] reported the impact of the ‘PhoenixCare’ intervention, [30] which provided integrated case-management for patients with either COPD or CHF who had an estimated life expectancy less than two years. The service was developed and overseen by a palliative care provider, but operated within the patients' managed care organisation (MCO). A nurse case-manager, supported by a team (medical director, social worker, counsellor) provided care which had four foci: self-management of illness and knowledge of resources, preparation for end of life, physical and mental functioning, and utilisation of medical services. The control group received ‘usual care’ which could include case-management according to normal practice in the MCO.
- Egan *et al.* [28] reported a hospital-based intervention, comprising respiratory nurse-led case-management of patients during and after an admission with an exacerbation of COPD in a private hospital in Brisbane, Australia. The intervention included comprehensive assessment at admission, a pre-discharge case-conference, and telephone follow-up at one and six weeks post-discharge. All four aspects of holistic care were well addressed. Patients in the control group received usual care with no contact with the case-manager, no case-conferences, and no post-discharge follow-up.
- Noonill *et al.* [29] described ‘Community Care for COPD’, a community-based intervention within tambons (administrative sub-districts of Thailand). The concept was developed from community-based pulmonary rehabilitation and promotion of exercise was an important focus of both the intervention and outcomes. Delivery was nurse-led and embraced co-ordination of care for patients with severe COPD, underpinned by a theoretically-based intervention, [31] which focussed on mobilisation of community resources including recruiting lay community health volunteers, systematic education and integrating positive health changes into lifestyle. Apart from spiritual support, all other components of holistic care were well addressed. The control group of patients had usual care; described as (often distant) hospital-based acute care with ‘limited chronic care for COPD patients’.

### Methodological quality

The results of the quality assessment are detailed in Table 2. Concerns about selective reporting of outcomes (and domains within patient-reported outcomes) resulted in Aiken 2006 being judged at moderate risk of bias. [27] Egan 2002 was judged at high risk of bias because of lack of information about blinding of researcher, and the handling of incomplete datasets. [28] Noonill *et al.* was, after substantial clarification by the authors of the process of randomisation and blinding of the researchers, judged at moderate risk of bias. [29]

**Table 2:** Risk of bias in included studies.

Study, year	Design	Adequate sequence generation	Allocation concealment	Blinding of research personnel‡	Blinding of outcome	Incomplete outcome data addressed	Free of selective reporting	Free of other bias	Risk of bias	Notes/limitations
Aiken *et al.* [27]	RCT	Yes Sealed envelopes, assigned in order of shuffling, stratified by diagnosis	Yes	Yes	Yes. Telephone interviews administered by a blinded professional interviewing firm.	Yes, though not reported for COPD sub-group. Overall loss of 66% of intervention and 75% of control participants by 9 months. ‘As many cases as possible were included in the analysis’	No Only a subset of data (56/112 questions+use of healthcare resources) reported. Different measures reported at different time points.	Yes	**Moderate**	Limited COPD data; small sub-group sample.
Egan *et al.* [28]	RCT	Yes Random number tables, stratified by severity	Unclear	Not reported	Not reported	Not reported	Yes	Yes	**High**	Small sample size, unclear reporting. Attrition due to death – unclear how many from each group
Noonill *et al.* [29]	CCT	Yes. Sealed envelopes*	Yes* COPD patients ‘were recruited from 6 tambons. These tambons were then randomly assigned’	Yes* ‘The researcher who collected outcome data were blinded’	Not reported	Yes	Yes	Yes	**Moderate**	No allowance for clustering effects. Authors stated they were unaware of any cluster effects (though original data no longer available)

* Clarified by author on request.

‡ Blinding of participants not possible.

### Effectiveness of interventions

The findings of the studies are detailed in Table 3.

**Table 3:** Main findings and interpretation.

	Health-related quality of Life	Measures of physical, psychological, spiritual and social well-being	Health and/or social service resource use	Satisfaction with care	Interpretation
Aiken *et al.* [27]	SF-36 mean differences not reported for COPD sub-group. Growth curve modeling of functional status over time was reported for domains of SF-36 for the COPD sub-group. Physical functioning at baseline: 13.0. change at 9 months: intervention +1.00 vs control −0.95 p<0.05. General health at baseline: 17.4. change at 9 months: intervention: +0.54 vs control −1.67 p<0.05. No significant differences in the other domains.	2 (of 11) non-validated questions with significant differences at 3 months (no difference at 6 months) were reported for COPD sub-group: ‘Begun or resumed an enjoyable activity in the previous 4 weeks’ Intervention 63% vs control 16% p = 0.01. ‘Experienced an event in the previous 4 weeks for which he/she felt unprepared’ Intervention 32% vs control 58% p<0.05 Mean frequency, severity and distress of the most troublesome symptom (part of the MSAS) was reported for COPD patients: Mean symptom distress was significantly lower in the intervention group than in controls at 3 months (mean 3.41 vs 4.29 on a 5-point scale, p<0.05). No significant difference at 6 months, or for frequency or severity of symptom at either time point	Medical system utilization (including hospitalisation) not reported for the COPD sub-group, though there was no difference in the combined group	Number of months in programme not reported for COPD patients, though attrition in the combined group is reported as ‘At the end of data collection 44% of the PhoenixCare participants and 25% of control patients were still participating’	There was some evidence that the PhoenixCare intervention had a small, transient effect on selected domains of quality of life and distress due to breathlessness.
Egan *et al.* [28]	No significant difference in the SGRQ at 1 month: Median change intervention: −1.6 vs control −1.5 p = 0.621	There were no significant differences in SWB (Median change intervention: 2.8 vs control −2.8 p = 0.416), HADS anxiety (Median change intervention: −1.0 vs control −2.5 p = 0.437) HADS depression (Median change intervention: 0.5 vs control −1.0 p = 0.383). The ‘affectionate support’ domain of the SSS showed a significant difference (Median change intervention: −6.7 vs control 0.0 p = 0.034	The mean number of unscheduled readmissions for the intervention group patients was 2.1 and for control group patients was 2.6	Qualitative data suggested the intervention was perceived to improve access to resources and communication (staff-patient and staff-staff).	Case management of inpatients did not improve quality of life, or anxiety and depression
Noonill *et al.* [29]	SGRQ at 3 months: intervention: 30.3 (SD 19.4) vs control 52.4 (21.3) p<0.001. Scores for control group at baseline not given. Improvement of 20.1 in total SGRQ score (minimal clinical important difference 4)	6MWD at 3-months: intervention: 342.8 (106.1) vs control: 265.1 (94.4) p = 0.001. Dyspnoea VAS at 3-months: intervention: 4.5 (2.2) vs control: 6.2 (1.8) p = 0.000	Hospitalisation in previous 3-months 3/43 (7.0) 2/44 (4.5) p = 0.651	PSCQ at 3 months (intervention: 91.1 (10.7) vs control: 74.9 (15.4) p<0.001)	When compared to the limited community care available to people with COPD in Thailand, the ‘Community Care for COPD’ intervention' resulted in highly significant improvements in quality of life, breathlessness, exercise tolerance, though no impact on hospitalisation. [No allowance for clustering effects]

6MWD - Six-minute walk distance measures the distance a patient can walk quickly on a flat, hard surface in 6 minutes reflects ability to perform daily activities, [34]

Dyspnoea VAS - Dyspnoea Visual Analog Scale, measures breathlessness in the sensory-perceptual domain [35].

HADS – Hospital Anxiety and Depression Score. Scores ≥11 indicate significant anxiety or depression; ≤7 are normal. [53]

MSAS - Memorial Symptom Assessment Scale assesses symptom prevalence, characteristics and distress. [36]

PSCQ - Patient satisfaction with care questionnaire reflects satisfaction with six aspects of care: technical quality, interpersonal manner, communication, financial aspects of care, time spent with doctor, and accessability of care. [38]

SF-36 - Medical Outcomes Study Short-Form-36 Health Survey is a generic health status measure with two summary measures of physical and mental health constructed from the eight scales. [33]

SGRQ – St George's Respiratory Questionnaire measures symptoms, activities and impacts on a scale: 0 to 100 (greatest impairement); with a minimum clinically important difference of 4). [32]

SSS - Social Support Survey has four functional support scales (emotional/informational, tangible, affectionate, and positive social interaction) and the construction of an overall social support index. [37]

SWB - Subjective Well-Being Scale is a longitudinal measure of the quality of life of patients with metastatic, incurable cancer. [54]

#### Health-related quality of life

The impact on quality of life varied in the three trials. Noonill *et al.* [29] reported that at three months HRQoL measured with the St George's Respiratory Questionnaire (SGRQ) [32] was significantly better in the patients from the intervention compared to control tambons, though no allowance was made for cluster effects (intervention group: 30.3 (19.4), control group: 52.4 (21.3) p<0.001). [29] This difference is substantially greater than the minimum clinically important difference for the SGRQ of four. [32] Aiken *et al.* reported that the rate of decline over nine months in two domains (‘Physical functioning’ and ‘General health’) of the Medical Outcomes Study Short-Form-36 Health Survey (SF-36) [33] was less in the PhoenixCare group than in the control group, though there was no significant difference in the other domains, or at 6-months. [27] Egan *et al.* observed no significant difference in the SGRQ at one month (Median change: intervention: −1.6 vs control −1.5 p = 0.621) as a result of in-patient case-management. [28]

#### Other outcomes: measures of physical, psychological, spiritual and social well-being

The outcome measures reported by Noonill *et al.* reflect the exercise component of the ‘Community Care for COPD’ and significant improvement was seen at 3-months in both the 6-minute walking test (6MWD) [34] (intervention: 342.8 (106.1) vs control: 265.1 (94.4) p = 0.001) and the Dyspnoea Visual Analog Scale [35] (intervention: 4.5 (2.2) vs control: 6.2 (1.8) p = 0.000). Aiken *et al.* reported that mean symptom distress (part of the Memorial Symptom Assessment Scale [36]) was significantly lower in the PheonixCare intervention group than in controls at 3-months (mean 3.41 vs 4.29 on a 5-point scale, p<0.05). There was no significant difference at 6-months, or in the frequency or severity of symptoms at either time point. [27]

Psychological well-being was reported by Egan *et al*. [28], who reported no significant differences in subjective well-being, or anxiety and depression at one month. The ‘affectionate support’ domain of the Social Support Survey [37] however showed a significant difference (Median change intervention: −6.7 vs control 0.0 p = 0.034). Aiken *et al.* [27] used 11 non-validated questions to assess the impact of the PhoenixCare intervention on self-management of illness and resumption of activities, but only reported two for the COPD sub-group which showed small significant differences at 3-months, but which were not sustained to 6-months.

#### Health and/or social service resource use

None of the trials showed a difference in health service utilisation, [27], [28], [29] though Aiken *et al*. did not report data for the COPD sub-group. [27]

#### Satisfaction with care

Noonill *et al.* [29] measured patient satisfaction with the validated Patient Satisfaction with Care (PSCQ). [38] At 3-months patients in the tambons which received the ‘Community Care for COPD’ intervention were significantly more satisfied than the controls (intervention: 91.1 (10.7) vs control: 74.9 (15.4) p<0.001). [29] Egan *et al.* undertook a nested qualitative study and concluded that there was a perceived improvement in access to resources and communication which enhanced patient care. [28]

## Discussion

We found three controlled trials of holistic interventions designed to deliver or enhance supportive care for people with severe COPD. All the interventions involved nurse-led case-management/co-ordination roles. The two RCTs, however, were either small studies, [28] included COPD as a sub-group, [27] were at risk of bias because of selective reporting, [27] or lack of blinding of researchers. [28] The CCT of co-ordinated community support in Thailand showed the most consistent effect (but follow up was only three months) in terms of improved HRQoL, exercise tolerance, dyspnoea, and satisfaction with care compared to the very low level of support available to the control group (though no allowance was made for cluster effects). [29] The impact of the PhoenixCare intervention in the American trial was confined to sub-domains of outcome measures and transient: [27] the Australian intervention showed no significant benefit. [28] There is, thus, a lack of robust evidence to inform the design of holistic interventions to support people living with severe COPD.

### Strengths and limitations

We searched a broad range of published and unpublished sources, and reassuringly no further studies were highlighted by the panel of international experts. Nevertheless we may have overlooked some relevant work. Our focus on RCTs meant that we did not, for example, include observational studies. We would have missed trials published prior to 1990, though this is unlikely as observational and qualitative studies identifying the problem only appear in the literature from the late 1990s. [2], [6], [13] We excluded studies that provided holistic care for patients with a range of advanced non-malignant disease, unless they reported outcomes for a sub-group people with COPD: some of these studies might have been informative though they could not address our specific question. We imposed no language or geographical restrictions on included studies.

A key challenge for the review was the definition of a ‘holistic intervention’. We defined this as an intervention which included at least three of the components of supportive care (physical, psychological, social and spiritual), but recognised that the tight word count of an abstract might preclude adequate description. In order not to risk rejecting potentially eligible studies at abstract stage we therefore reviewed the full paper if the abstract alluded to two or more domains, only excluding them if the full text did not explicitly describe three domains.

### Interpretation of findings in relation to previously published work

The common concept underpinning the interventions described in the three papers included in our review was the nurse-led co-ordination of services described in two of the studies as ‘case-management’. [28], [29] Interpretation of this concept varied according to context. In the USA, the PhoenixCare Programme integrated with the existing MCOs: an approach which has been shown to improve HRQoL in the frail elderly, [39], [40], [41] improve patient satisfaction in groups with a range of life-threatening conditions, [42], [43] though impact on use of healthcare resources is variable. [44] In Thailand, the study which showed the most consistent improvement, the nurse co-ordinator not only provided individual specialist care for patients but also catalysed lay community support which aimed to encourage greater levels of activity by integrating people potentially isolated by their breathlessness into the community. The role of the voluntary sector may have relevance for other societies in the form of local patient support groups, [45] and more broadly in terms of community support. [46]

The impact of a complex intervention depends as much on the nature of the care provided to the control group as on the effectiveness of the intervention. In Thailand, where the usual care was very limited, [29] the intervention had a substantial impact (on activities and quality of life, though not admissions) whereas comparison with the care provided by an MCO which could include case-management may offer less scope for improvement. [27]

### Implications for policy, practice and research

A key consideration for developing and evaluating interventions is the choice of outcome measures. [47] Costly admissions may be the outcome of interest to healthcare planners, whereas in order to assess the wider impact of an intervention, HRQoL may be more appropriate. Specific symptoms such as breathlessness may be a key problem, [19] but for a potentially isolated housebound patient the resultant social concerns may be paramount. The range of outcomes used both in the studies we reviewed and more widely in the literature on management of severe COPD, [48], [49], [50], [51], [52] may be symptomatic of the challenge of evaluating a broad holistic intervention as effects may be expected to be diverse and the most appropriate primary outcome may not be apparent. There may be value in reaching consensus about core outcomes for trials in this area in order to facilitate future synthesis of evidence.

## Conclusions

Some 15 years after the early reports highlighting the unmet needs of people with severe COPD, [2], [13] we have been unable to find robust evidence about interventions which can address those needs. Globally, over the next two decades the number of people living with, and potentially dying from COPD is predicted to increase substantially, [1] but healthcare services seeking to develop services to meet this challenge currently have little evidence on which to base their decisions. There is, thus, an urgent need rigorously to develop and evaluate interventions designed to deliver or enhance holistic care and improve quality of life of people with severe COPD.

## Supporting Information

Table S1 **Search strategies.**
(DOCX)

Table S2 **List of experts contacted.**
(DOCX)
