# Supplementary material for: Effectiveness of Holistic Interventions for People with Severe Chronic Obstructive Pulmonary Disease: Systematic Review of Controlled Clinical Trials
Source: PLoS One. 2012 Oct 23;7(10):e46433. doi: 10.1371/journal.pone.0046433 (PMC3479091; doi:10.1371/journal.pone.0046433)
Supplement: Table S1 — Search strategies. (DOCX) [file pone.0046433.s001.docx]

**Table S1: Search strategies**

| **Search strategies for MEDLINE, EMBASE, AMED and PsycINFO** |
| --- |
| 1. exp Intervention Studies/ 2. intervention studies.mp. 3. experimental stud*.mp. 4. exp Clinical Trial/ 5. clinical trial.mp. 6. exp Controlled Clinical Trial/ 7. controlled clinical trial.mp. 8. exp Randomized Controlled Trial/ 9. randomized controlled trial.mp. 10. randomi* controlled trial.mp. 11. quasi-randomi* controlled trial.mp. 12. non-randomi* trial.mp. 13. exp Placebos/ 14. placebos.mp. 15. exp Random Allocation/ 16. random allocation.mp. 17. exp Double-Blind Method/ 18. double-blind method.mp. 19. double-blind design.mp. 20. exp Single-Blind Method/ 21. single-blind method.mp. 22. random*.mp. 23. or/1-22 24. exp Holistic Health/ 25. holistic.mp. 26. exp Palliative Care/ 27. paliative care.mp. 28. exp "Quality of Life"/ 29. quality of life.mp. 30. health related quality of life.mp. 31. physical well-being.mp. 32. psychological well-being.mp. 33. spiritual well-being.mp. 34. exp "Quality of Health Care"/ 35. exp Quality Assurance, Health Care/ 36. exp Quality Indicators, Health Care/ 37. exp Long-Term Care/ 38. quality of care.mp. 39. (Social and Healthcare Support).mp. 40. or/24-39 41. "severe chronic obstructive pulmonary disease".mp. 42. severe COPD.mp. 43. “end-stage chronic obstructive pulmonary disease”.mp. 44. “end-stage COPD”.mp. 45. “advanced chronic obstructive pulmonary disease”.mp. 46. “advanced COPD”.mp. 47. or/41-47 48. 23 AND 40 AND 47 49. limit 48 to yr="1990 - Current" |
| **Search strategy for The Cochrane Library, ISI Web of Science, CINAHL, LILACS, ZETOC, DARE and British Nursing Index** |
| (“severe chronic obstructive pulmonary disease” or “severe COPD” or advanced chronic obstructive pulmonary disease or end-stage chronic obstructive pulmonary disease or obstructive lung disease* or obstructive pulmonary disease* or pulmonary emphysema or chronic bronchitis)  AND  (intervention stud* or experimental stud* or clinical trial or controlled clinical trial or randomised controlled trial or quasi-randomised clinical trial)  AND  (“holistic health” or “palliative care” or “long-term care” or “quality of life” or “health related quality of life”) |
