# Supplementary material for: Effectiveness of Holistic Interventions for People with Severe Chronic Obstructive Pulmonary Disease: Systematic Review of Controlled Clinical Trials
Source: PLoS One. 2012 Oct 23;7(10):e46433. doi: 10.1371/journal.pone.0046433 (PMC3479091; doi:10.1371/journal.pone.0046433)
Supplement: Table S2 — List of experts contacted. (DOCX) [file pone.0046433.s002.docx]

**Table S2: List of experts contacted**

| **Name of author** | **Country** | **Email address** |
| --- | --- | --- |
| Julia Addington-Hall | UK | J.Addington-Hall@soton.ac.uk |
| Claudia Bausewein | UK | Claudia.Bausewein@kcl.ac.uk |
| David Currow | Australia | David.Currow@rgh.sa.gov.au |
| J Randall Curtis | USA | jrc@u.washington.edu |
| John Ellershaw | UK | Jellershaw@mariecurie.org.uk |
| Morag Farquhar | UK | Mcf22@medschl.cam.ac.uk |
| Donna Goodridge | Canada | Donna.Goodridge@usask.ca |
| Irene Higginson | UK | Irene.Higginson@kcl.ac.uk |
| Geoffrey Mitchell | Australia | g.mitchell@uq.edu.au |
| Eric van Rijswijk | The Netherlands | Evanrijswijk.huisarts@gmail.com |
| Graeme Rocker | Canada | Graeme.Rocker@Dal.Ca |
| David Seamark | UK | cjseamark@doctors.org.uk |
| Nils Schneider | The Netherlands | Schneider.Nils@MH-Hannover.de |
